# In Silico Identification of 2,4-Diaminopyrimidine-Based Compounds as Potential CK1ε Inhibitors

**DOI:** 10.3390/ph18050741

**Published:** 2025-05-17

**Authors:** Axel A. Sánchez-Álvarez, Marco A. Velasco-Velázquez, Luis Cordova-Bahena

**Affiliations:** 1 School of Medicine, Universidad Nacional Autónoma de México (UNAM), Mexico City 04510, Mexico; aaalvarez@comunidad.unam.mx; 2 Graduate Program in Chemical Sciences, Universidad Nacional Autónoma de México, Mexico City 04510, Mexico; 3 Secretaría de Ciencia, Humanidades, Tecnología e Innovación (SECIHTI), Mexico City 04510, Mexico

**Keywords:** CK1ε, cancer, virtual screening, diaminopyrimidine, molecular dynamic simulations, pharmacophore model

## Abstract

**Background:** Casein kinase 1 epsilon (CK1ε) plays a critical role in cancer progression by activating oncogenic signaling pathways, making it a target for cancer therapy. However, no inhibitors are currently available for clinical use, highlighting the need for novel therapeutic candidates. **Methods:** This study aimed to identify potential CK1ε inhibitors. To achieve this, a modified version of a previously reported pharmacophore model was applied to an ultra-large database of over 100 million compounds for virtual screening. Hits were filtered based on drug-likeness and pH-dependent pharmacophore compliance and then grouped according to their structural core. A representative compound from each structural group underwent molecular dynamic (MD) simulations and binding free energy calculations to predict its stability and affinity, allowing extrapolation of the results to the entire set of candidates. **Results:** Pharmacophore matching initially identified 290 compounds. After energy minimization, and an assessment of drug-likeness and pharmacophore compliance, we selected 29 structurally related candidates. MD simulations showed that most of the compounds representative of structural groups had stable binding modes, favorable intermolecular interactions, and free energies comparable to those of previously reported CK1ε inhibitors. An analysis of additional members of the most promising structural group showed that two 2,4-diaminopyrimidine-based compounds likely inhibit CK1ε. **Conclusions:** These findings provide structural insights into the design of CK1ε inhibitors, supporting compound optimization and the eventual development of targeted cancer therapeutics.

## 1. Introduction

**Figure 1:**
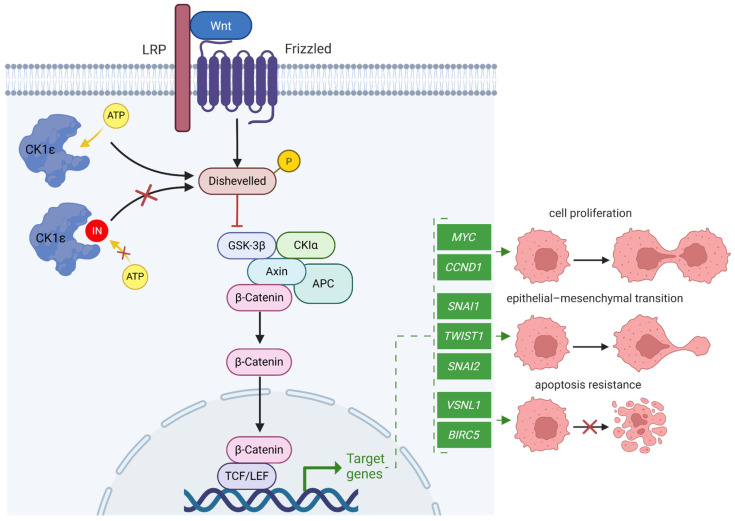
Schematic representation of CK1ε’s role in the Wnt/β-catenin signaling pathway. Proteins are represented as ellipses, with CK1ε highlighted by a distinct shape. Target genes are represented as green rectangles. ATP and a competitive inhibitor are shown as yellow and red circles, respectively. Solid arrows indicate downstream signaling effects. Dashed arrows represent cellular outcomes resulting from the regulation of target genes.

Casein kinase 1 (CK1) is a family of serine/threonine protein kinases involved in key cellular processes, including cell cycle regulation, apoptosis, and DNA repair [1,2]. Among its isoforms, CK1ε plays a crucial role in maintaining physiological functions, such as circadian rhythm regulation, neuronal signaling, and immune response modulation [3,4,5]. In cancer, CK1ε is a positive regulator of the Wnt/β-catenin pathway by phosphorylating Disheveled (DVL), leading to β-catenin stabilization and the subsequent activation of Wnt target genes [6,7,8]. Those genes promote tumor progression by driving uncontrolled proliferation [9,10], epithelial-to-mesenchymal transition [11,12], and resistance to apoptosis [13,14,15,16] (Figure 1). Accordingly, CK1ε overexpression has been implicated in various cancer types [17,18]. Recent studies have provided quantitative evidence of its upregulation in several malignancies, reinforcing its relevance as a therapeutic target. CK1ε expression has been reported in breast cancer samples, with elevated levels shown to correlate with improved disease-free survival in specific patient cohorts [19]. Notably, higher CK1ε levels correlate with improved disease-free survival in patient subsets, suggesting a context-dependent role of CK1ε in tumor biology [19]. In breast cancer, CK1ε enhances migration and invasion, particularly in triple-negative subtypes, contributing to aggressive tumor behavior [20,21,22]. In colorectal cancer, CK1ε is significantly upregulated in tumor tissues compared to adjacent normal tissues. This overexpression is associated with advanced tumor stages and poorer overall survival, underscoring its potential role in tumor progression and as a prognostic marker [6,23,24]. In colorectal cancer, CK1ε-dependent β-catenin activation correlates with poor prognosis and enhanced tumor growth [17,25]. In ovarian cancer, high CK1ε levels are associated with enhanced proliferation and resistance to apoptosis [26]. Additionally, CK1ε has been linked to glioblastoma progression, where it facilitates the self-renewal of cancer stem-like cells and resistance to therapy [27,28].

The key role of CK1ε in oncogenic signaling has driven the development of selective inhibitors of its catalytic activity. For example, PF-4800567 (designated as reference 1 [REF 1] from herein) has been co-crystallized with CK1ε [29]. It exhibits an IC₅₀ of 32 nM against CK1ε in kinase assays. A second inhibitor, SR-4133 (REF 2), is a potent (IC₅₀ = 15 nM) CK1ε inhibitor with reduced off-target activity due to increased selectivity [30]. For example, the IC₅₀ value for inhibiting casein kinase 1 delta (CK1δ) is more than 10-fold higher than for CK1ε [30]. A third inhibitor, Umbralisib (REF 3), targets both PI3Kδ and CK1ε, with an IC₅₀ of 37 nM against the latter [31]. This inhibitor of CK1ε is effective in reducing tumor growth in preclinical models of lymphoma [31]. Umbralisib was approved by the FDA in 2021 for the treatment of relapsed or refractory marginal zone lymphoma and follicular lymphoma [32]. However, it was withdrawn in 2022 after updated data from the UNITY-CLL clinical trial (NCT02612311) revealed an increased risk of death in patients receiving it [33].

Despite the success of several marketed kinase inhibitors in cancer therapy, their clinical use remains challenged by issues such as limited selectivity, off-target effects, and acquired resistance [34]. Many of these inhibitors share conserved scaffolds that interact with the ATP-binding site, which is highly similar across kinase families, thereby increasing the risk of cross-reactivity [35]. These limitations underscore the need for novel or repurposed compounds capable of achieving selective engagement with specific kinases, such as CK1ε. In this context, the identification of ligands that exploit less conserved regions or unique interaction patterns offers a promising strategy to improve therapeutic precision.

The structure of CK1ε consists of an N-lobe rich in β-sheets and a C-lobe composed of α-helices, connected by a hinge region that forms the ATP-binding site [36]. This pocket is divided into key subsites that contribute to ligand binding and selectivity. The adenine-binding region stabilizes interactions of both the substrate and inhibitors through hydrogen bonds with E83 and L85. A deep hydrophobic pocket accommodates nonpolar moieties. Finally, a phosphate-binding region interacts with charged groups, playing a critical role in ATP recognition [37]. These structural features can be exploited for the development of selective inhibitors with enhanced affinity for CK1ε.

In this study, we employed a virtual screening protocol aiming at the identification of potential CK1ε inhibitors. Using a previously reported pharmacophore [38] and analyzing drug-likeness and pH-dependent pharmacophore compliance, we screened a database with 103,302,052 compounds. Hits were grouped by structural similarity and the interaction of a representative compound of each group with CK1ε was studied by molecular dynamic (MD) simulations. We found that most of the compounds maintained stable binding modes, exhibited favorable intermolecular interactions, and showed binding affinities comparable to those of reference inhibitors. A further analysis of compounds within the most promising structural group identified two 2,4-diaminopyrimidine-based compounds as potential CK1ε inhibitors. These results offer valuable structural insights for the rational design of CK1ε inhibitors and lay the groundwork for future optimization and therapeutic development.

## 2. Results

### 2.1. Identification of Compounds Matching the Pharmacophore Model

We previously developed a pharmacophore for targeting the ATP-binding site of CK1ε [38]. In such a model, the aromatic feature 1 (Aro1) and the hydrophobic feature 1 (Hyd1) overlap in space. For this work, we removed the Hyd1 feature since it excludes compounds containing heterocycles even though they can form stacking interactions with the target residue (F20). This simple modification aimed to expand the structural diversity of the pharmacophore-matching compounds

The modified pharmacophore was employed via the Pharmit server (https://pharmit.csb.pitt.edu (accessed on 21 March 2025)) [39], for the screening of the PubChem database [40]. As a result, we identified 290 different compounds with the potential to bind the ATP-binding site of CK1ε. On the other hand, the original pharmacophore model retrieved 236 hits. Removing the Hyd1 feature increased the hit rate by 22.88%, while preserving high structural diversity among the identified compounds (Figure S1).

**Figure 2:**
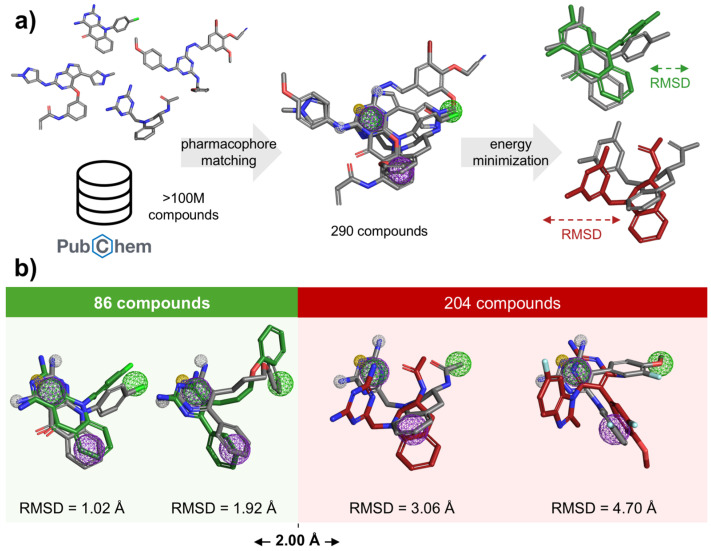
Compound identification and RMSD analysis after energy minimization. (**a**) Identification of pharmacophore-matching compounds and their energy-minimized conformers within the ATP-binding site. (**b**) Pharmacophore matching (gray) vs. minimized conformers (colored). Compounds with an RMSD >2.0 Å (red chart) were discarded. Pharmacophore features: purple (aromatic), yellow (hydrogen bond acceptor: HBA), white (hydrogen bond donor: HBD), and green (hydrophobic).

Refinement of the selection included a comparison of the pharmacophore-matching conformations with those generated through energy minimization in the context of the target protein. The latter were generated using the Smina scoring function [41], which accounted for both intermolecular and intramolecular contributions. To prioritize compounds closely aligned with the pharmacophore within the ATP-binding site, we applied a root-mean-square deviation (RMSD) threshold of ≤2.0 Å following energy minimization. This criterion aimed to retain conformers with minimal structural distortion from the original pharmacophore-matching conformer, ensuring compatibility with the binding site geometry. While this threshold may exclude ligands capable of adopting favorable poses upon relaxation, it was chosen to preserve the dataset with candidates that align to the hypothesized binding mode. As these results suggest that the pharmacophore-matching conformation is not energetically favored, those compounds were excluded from the further analysis. On the other hand, 86 compounds showed an RMSD ≤ 2.0 Å and were still considered candidates (Figure 2).

### 2.2. Cheminformatic Analysis 

To assess the drug-likeness, we calculated the molecular weight, number of hydrogen bond acceptors and donors, and the Moriguchi octanol–water partition coefficient for the 86 compounds selected through energy minimization (Table S1). Of those, only 66 compounds displayed no violations of Lipinski’s Rule of Five (Ro5) and were prioritized since they may have favorable bioavailability after oral administration [42].

The pharmacophore query included two hydrogen bond donor (HBD) and one acceptor (HBA) features arranged in an HBD–HBA–HBD configuration. Since the compliance with this configuration depends on the protonation state of the candidate molecules, we calculated the distribution of microspecies as a function of the pH for the 66 compounds that complied with Lipinski’s rules. At pH 7.4, only 29 compounds exhibited ≥ 90% of microspecies matching the required configuration (Table S2) and were considered the compounds of interest.

To assess potential selectivity, the 29 compounds of interest were scored against CK1ε and its closest homolog, CK1δ (PDB 4HNF). All compounds exhibited favorable binding scores that were comparable between CK1ε and CK1δ, with no clear selectivity trends observed (Table S3).

### 2.3. Framework-Based Clustering

**Figure 3:**
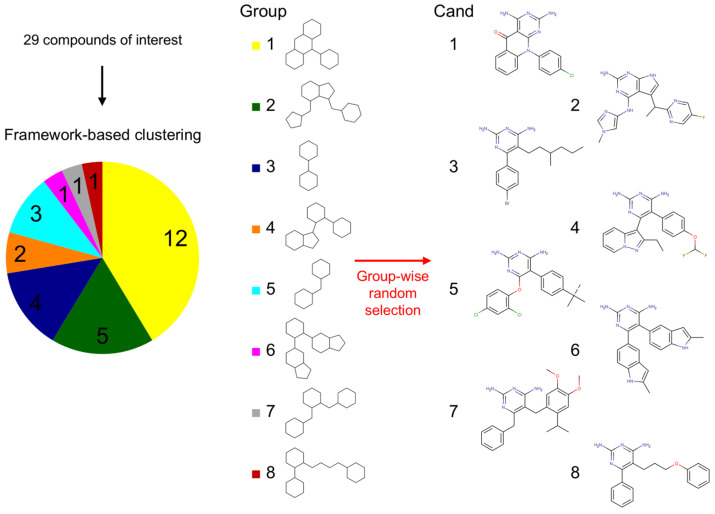
Clustering of compounds based on Bemis & Murcko (B&M) frameworks. The compounds of interest were divided into eight groups based on shared structural frameworks. The numbers in the pie graph show the number of compounds per group. Each B&M framework is labeled with its corresponding group number and color-coded to match the pie graph. A randomly selected candidate (Cand) for MD studies from each cluster is displayed as a 2D structure.

To broadly explore the chemical space among potential CK1ε inhibitors, the Bemis & Murcko (B&M) frameworks of the compounds of interest were determined to identify the core structural features. The 29 compounds of interest were clustered into groups based on their shared framework consisting of ring systems and their linkers, with all bonds treated as single and substituents omitted. We identified eight groups, with compound counts ranging from 12 in group 1 to a single compound in groups 6 through 8. For subsequent analyses, we selected one representative compound per group to minimize redundancy and maximize structural coverage within the compounds of interest. Sampled compounds were designated as candidates (Cand) 1–8 according to their group of origin (Figure 3).

### 2.4. MD Simulations for Representative Compounds

MD simulations (200 ns) were performed to assess the dynamic behavior of complexes of Cand 1–8 with CK1ε. The minimized conformer from each group was used to form the CK1ε/cand 1–8 complexes. Two independent simulations per compound ensured result reliability, while binding mode stability was evaluated using RMSD calculations. 

**Figure 4:**
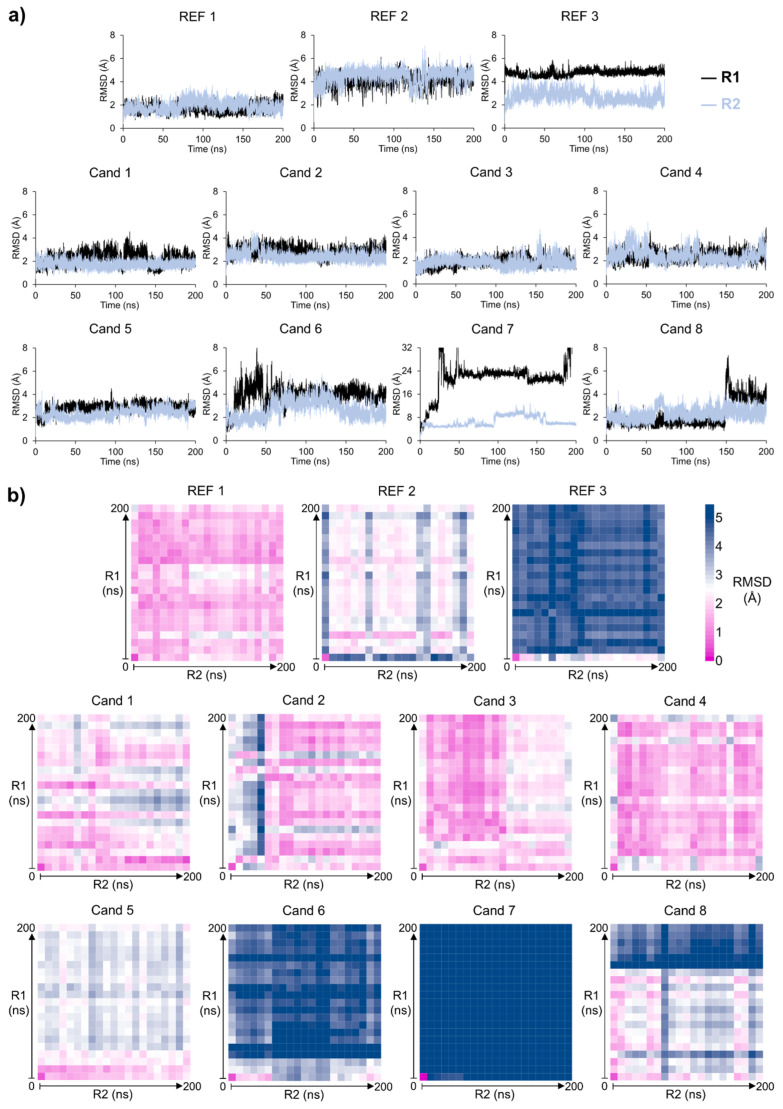
Molecular dynamic simulations of CK1ε with reference (REF) or candidate (Cand) compounds. (**a**) Heavy-atom RMSD of ligands across two independent replicates (R1 and R2). (**b**) RMSD matrices showing pairwise comparisons of ligand conformations between two independent replicates (R1 and R2) of CK1ε/ligand MD simulations. Axes represent simulation progression based on each replicate with ligand conformations sampled every 10 ns.

For a comparison, we also performed MD simulations for REF 1–3. REF 1, whose binding mode was previously determined via X-ray crystallography, did not show significant changes from the co-crystallized conformation. The binding behavior remained similar to the reported structure, with RMSD values of 1.68 ± 0.33 Å in the replica one (R1) and 1.91 ± 0.45 Å in the second one (R2), indicating high stability. Conversely, REF 2, whose binding mode was determined using a validated docking protocol [38], showed RMSD values of 4.16 ± 0.46 Å in R1 and 4.52 ± 0.60 Å in R2, suggesting a rearrangement to a distinct but stable conformation. Interestingly, REF 3, docked with the same protocol and exhibited RMSD values of 4.74 ± 0.27 Å in R1 and 2.68 ± 0.47 Å in R2. The solvent-exposed chromone and fluoromethyl rings of REF 3 underwent significant reorientation in R1 due to the rotation of their bond to the central core. In contrast, this reorientation was less pronounced in R2. A similar behavior was observed with the phenyl-isopropoxyphenyl group on the opposite side of the central core of REF 3. However, this group remained buried in the hydrophobic pocket, with reorientation less pronounced compared to the solvent-exposed moiety. Interestingly, the central core remained fixed throughout the entire molecular dynamic simulation. So, despite the deviation of specific motifs, the binding mode of REF 3 remained stable (Figure 4a).

Cand 1–5 displayed average RMSD values ranging from 1.75 to 2.83 Å, with standard deviations below 0.58 Å in replicates, indicating stable binding modes for all these compounds. In contrast, Cand 6 showed an RMSD of 3.63 ± 1.33 Å during the first 50 ns of R1, reflecting an unstable binding mode. However, from 50 to 200 ns, the RMSD stabilized at 3.98 ± 0.48 Å, suggesting that Cand 6 adopted a new stable conformation. This trend was not observed in R2, where the RMSD was 1.91 ± 0.39 Å during the first 50 ns, followed by a slight reorientation to stabilize it at 2.93 ± 0.68 Å in the final 150 ns, suggesting a difference between the binding modes reached at R1 and R2 (Figure 4a).

On the other hand, Cand 7 exhibited an RMSD of 17.99 ± 10.10 Å during the first 50 ns of R1, indicating that the ligand exited the ATP-binding site. Consistently, R2 of Cand 7 showed a RMSD of 6.56 ± 1.79 Å, confirming its instability (Figure 4a). Finally, Cand 8 showed RMSD values of 2.29 ± 1.08 Å in R1 and 2.31 ± 0.49 Å in R2, suggesting stable binding modes. Notably, the most pronounced conformational changes occurred toward the end of R1, indicating that Cand 8 may explore alternative conformations in longer simulations (Figure 4a).

The CK1ε backbone RMSD was analyzed to assess the possible changes in the stability of the protein. Across all simulations, the mean RMSD of the backbone ranged from 1.95 to 2.60 Å, with a maximum standard deviation of 0.42 Å (Table S4), indicating no relevant conformational changes in the target protein. Moreover, the dynamic cross-correlation maps (DCCMs) of the protein Cα atoms revealed consistently high positive correlations across residues within the N-terminal lobe and across residues within the C-terminal lobe (Figure S2). In contrast, the interconnecting loop exhibited uncorrelated movements with respect to both lobes, with a slightly stronger correlation with the C-terminal lobe. This behaviour indicates coordinated intra-lobe dynamics, accompanied by independent motion between the two lobes and the hinge region for all the complexes.

To assess whether the ligands explored similar conformational spaces, we generated conformational similarity matrices using RMSD values between conformations from R1 and R2, sampled every 10 ns from the full MD trajectories.

The resulting RMSD matrices revealed that complexes with REF 1, REF 2, and Cand 1–4 explored similar ligand conformations across both replicates. In contrast, REF 3, and Cand 6 showed significant differences (Figure S3) in ligand conformations between R1 and R2. Cand 5 exhibited distinct behavior, showing slight differences between R1 and R2 throughout the trajectories, which were attributed to the different orientations of the meta-dichlorobenzene ring bonded to the central core and exposed to the solvent. However, the central core remained fixed throughout the entire MD simulation, emphasizing the dynamic flexibility of the solvent-exposed moiety. Moreover, Cand 8 resembled REF 1 and REF 2 but adopted a unique conformation at 150 ns in R1. Finally, Cand 7 exhibited the greatest conformational differences across replicates, as expected from the comparison of two non-stable simulations, leading to its exclusion from further analysis (Figure 4b).

An assessment of residue fluctuations in CK1ε was evaluated by calculating the root mean square fluctuation (RMSF). The REF compounds influenced CK1ε similarly but showed differences at specific residues. F20 and G175, both located in the phosphate-binding region of the ATP-binding site, exhibited higher fluctuations with REF 2 compared to REF 1 and REF 3. CK1ε/REF 1 exhibited the lowest overall fluctuations compared to all other references and candidates. In contrast, CK1ε/REF 3 displayed higher fluctuations at S31 and G75 in the N-terminal lobe, as well as at residues 45–55 at the bottom of the druggable cavity (Figure S4).

All eight complexes with Cand 1 to 8 followed a similar fluctuation pattern with specific variations. F20 showed reduced fluctuations in complexes with Cand 1, 2, 3, and 5 compared to REF 2, but this reduction was not observed with candidates 4, 6, 7, and 8. G175 displayed lower fluctuations in complexes with Cand 3, 4, and 5, while Cand 1, 2, 6, 7, and 8 maintained higher fluctuations similar to REF 2. Additionally, S31 and G75 showed decreased fluctuations in complexes with Cand 2, 3, 4, 5, and 6 but remained higher in complexes with Cand 1, 7, and 8. Finally, residues 45–55 at the bottom of the cavity exhibited lower fluctuations across all Cand complexes compared to REF 3, indicating enhanced stabilization in this region. In general, the behavior of the candidates closely resembled that of REF 1. Notably, the reduced fluctuations observed in key residues, particularly at the bottom of the druggable cavity, indicate that most candidates promote a more stable protein–ligand interaction, potentially enhancing CK1ε binding affinity and complex stability (Figure S4).

**Figure 5:**
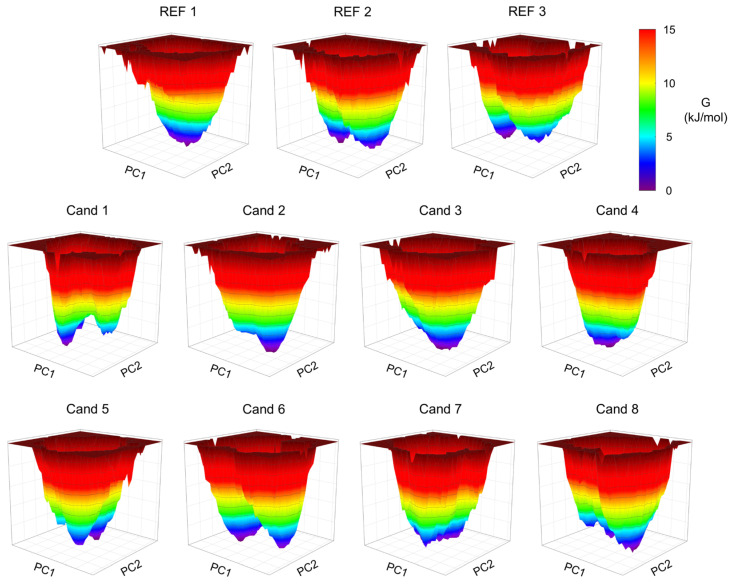
Principal-component-based free energy landscapes for CK1ε in complex with reference compounds and candidates. Binding free energy landscape (FEL) contour for the simulated CK1ε/REF1–3 and Cand 1–8 complexes.

To characterize the stable conformations adopted by the protein in response to ligand binding, free energy landscapes (FELs) were constructed based on the first two principal components (PC1 and PC2) of the protein dynamics during the simulations. Among the ligands, REF 1 and Cand 2–4 induced more rigid stabilization of the protein, as evidenced by a single pronounced energy minimum in their respective FELs. Interestingly, a similar behavior was observed for Cand 7. However, the single minimum in this case corresponded to that of the solvated protein in the absence of the ligand, as Cand 7 exited the binding site early in the simulation. In contrast, Cand 1, 5, and 8 showed multiple local minima, indicating a more flexible binding mode induced by these ligands. Similarly, REF 2, REF 3, and Cand 6 exhibited distinct stable protein conformations, but with more pronounced energy barriers between minima, suggesting more restricted transitions between states (Figure 5).

### 2.5. Binding Free Energy for the CK1ε/Candidate Complexes

**Figure 6:**
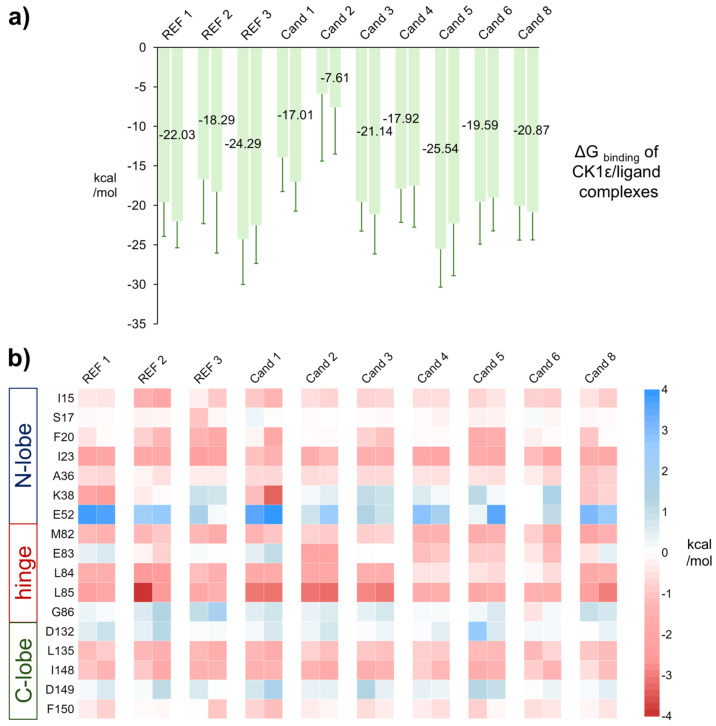
Binding energies of CK1ε/ligand complexes. (**a**) Free energy change (ΔG). The replicate with the lowest average ΔG is labeled accordingly. Error bars indicate the SD. (**b**) Per-residue decomposition of the enthalpic contribution. Two columns per compound are shown, each representing an independent MD replicate. The N-terminal lobe, hinge, and C-terminal lobe are indicated.

We then estimated the binding affinity of REF 1–3 and Cand 1–6 and 8 to CK1ε by using the Molecular Mechanics/Poisson–Boltzmann Surface Area-Interaction Entropy (MM/PBSA-IE) approach for each replicate. The average ΔG of REF 1–3 had values ranging from −17.29 to −25.29 kcal/mol. The ΔG of candidates ranged from −5.88 (Cand 2) to −25.54 kcal/mol (Cand 5) (Table S5). Cand 2 had the least favorable ΔG, primarily due to the entropic contribution, and was therefore excluded from further analysis. Notably, entropy penalties vary with ligand flexibility; flexible ligands typically incur greater entropy losses upon binding due to restricted conformational freedom, whereas rigid ligands experience smaller penalties. Cand 3 and 8 were the least affected by entropy despite having more rotatable bonds than the other ligands. Cand 5 exhibited the most favorable average ΔG among all the ligands (Figure 6a).

To identify key residues involved in ligand binding to CK1ε, we performed a per-residue decomposition of the enthalpic contribution. Independent replicates of CK1ε/ligand MD simulations were analyzed separately. Only amino acids within 6 Å of the ligand and showing a contribution exceeding ±1.0 kcal/mol in at least one complex were considered relevant.

All complexes exhibited similar per-residue contribution patterns, with some notable differences at specific residues. Hydrophobic residues I15, I23, A36, L135, and I148 consistently contributed favorably across all complexes. Similarly, M82, L84, and L85, located in the interconnection loop between the lobes, played a major role in stabilizing the binding mode, highlighting the importance of this loop for ligand binding. F20 also showed favorable contributions for most of the ligands, with stronger contributions in REF 2, REF 3, and Cand 3 and 5. In contrast, E52 consistently contributed unfavorably to the binding of all ligands, displaying greater values at REF 1 and Cand 1 complexes. Moreover, K38, located at the bottom of the cavity, displayed the highest variability, contributing favorably in REF 1, REF 2, and Cand 1 and 8 but unfavorably in REF 3 and Cand 2–6 (Figure 6b).

These findings highlight the key role of hydrophobic interactions in ligand binding to CK1ε, particularly involving residues I15, I23, A36, L135, and I148, which consistently contributed favorably. The variability observed at K38 suggests that specific ligands may interact differently in this region, influencing their binding affinity. Notably, the stabilization effect of M82, L84, and L85 in the interconnection loop underscores its importance in maintaining ligand interactions across all complexes. Overall, the binding profile of the candidates closely resembled that of REF 1, suggesting a conserved interaction pattern that may be relevant for future ligand optimization.

### 2.6. Analysis of the Binding Behavior

**Figure 7:**
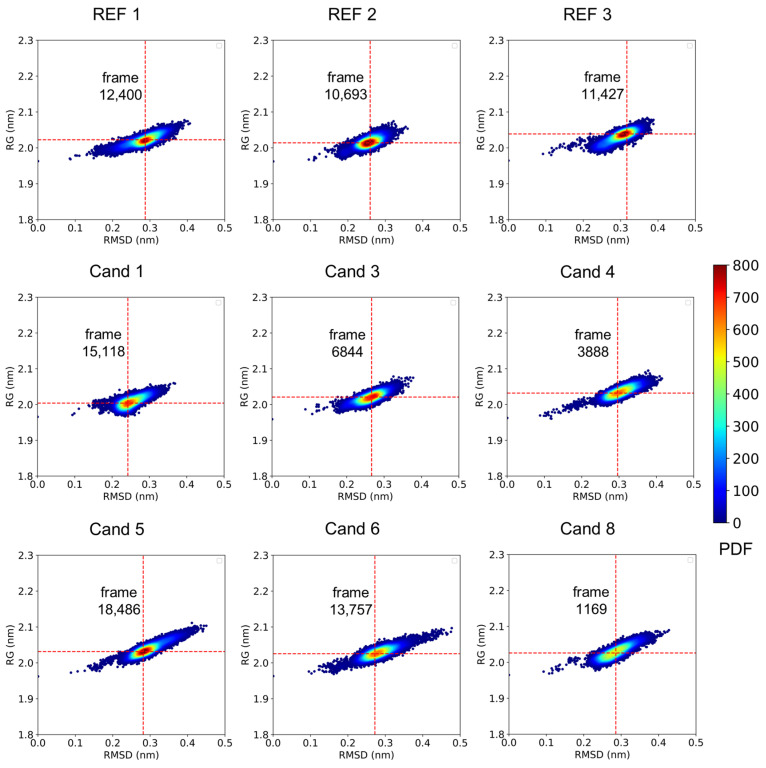
Probability density function plots for CK1ε/ligand complexes based on radius of gyration and RMSD. The sampled frame for each ligand representative binding mode is labeled accordingly, with red dashed lines indicating the associated Rg and RMSD values.

The most probable conformations of the CK1ε/ligand complexes were identified using a probability density function (PDF) constructed from the complexes’ radius of gyration (Rg) and RMSD. All complexes exhibited well-defined high density regions, with dominant conformations consistently clustered within the 1.9–2.1 nm range for Rg and 0.2–0.4 nm for RMSD (Figure 7). These findings suggest an overall structural similarity across the ensembles.

Given the structural consistency observed in the PDF analysis, a conformation from the highest-density region was sampled for each complex and designated as the representative binding mode.

**Figure 8:**
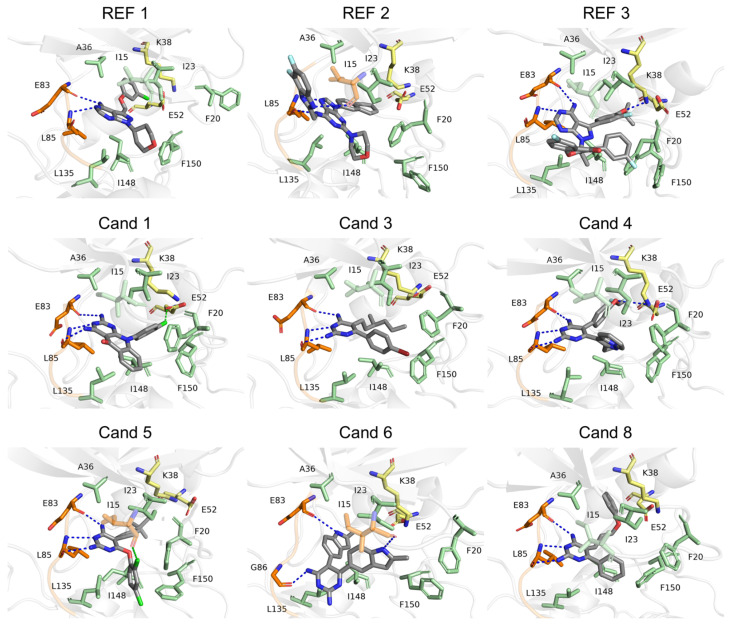
Representative binding modes of reference compounds and candidates. CK1ε is shown as a grey cartoon, with the hinge region colored orange. Ligands are depicted in grey. Interacting residues are shown as sticks: orange for residues involved in hydrogen or halogen bonds, green for those participating in hydrophobic interactions, and yellow for K38 and E52. Blue and green dashed lines indicate hydrogen and halogen bonds, respectively.

An analysis of CK1ε intermolecular interactions with REF and Cand ligands in their representative binding modes showed that binding was primarily driven by hydrogen bonds. These interactions mainly involved residues E83 and L85 in the hinge region of CK1ε during the MD simulations. While REF 1 and 3 and Cand 6 formed two hydrogen bonds with this region, REF 2 and Cand 1–5 and 8 established a hydrogen bond trident. This motif consists of a linear or fork-like arrangement of hydrogen bond donors and acceptors, where a central hydrogen bond acceptor (HBA) is flanked by two hydrogen bond donors (HBDs), resulting in an HBD–HBA–HBD configuration. The trident exhibited by REF 2 was derived from an HBD–HBD-HBA configuration and occurred mostly with Leu85. Differently, the trident formed by Cand 1–6 was derived from the HBD–HBA–HBD configuration, targeted by the pharmacophore model. Additionally, all ligands engaged in hydrophobic interactions with I15, F20, I23, A36, K38, and E52 in the N-terminal lobe of CK1ε and with L135, I148, and F150 in the C-terminal lobe. Additionally, Cand 6 formed one more hydrogen bond with I15. Interestingly, Cand 5 established a halogen bond with I15. Interestingly, residues K38 and E52 play distinct roles across different complexes. While K38 forms a hydrogen bond with REF 3 and Cand 4, E52 forms a halogen bond with Cand 1. In the other complexes, K38 and E52 remain available to form a salt bridge with each other (Figure 8).

### 2.7. Assessment of the Group 5 Members

**Figure 9:**
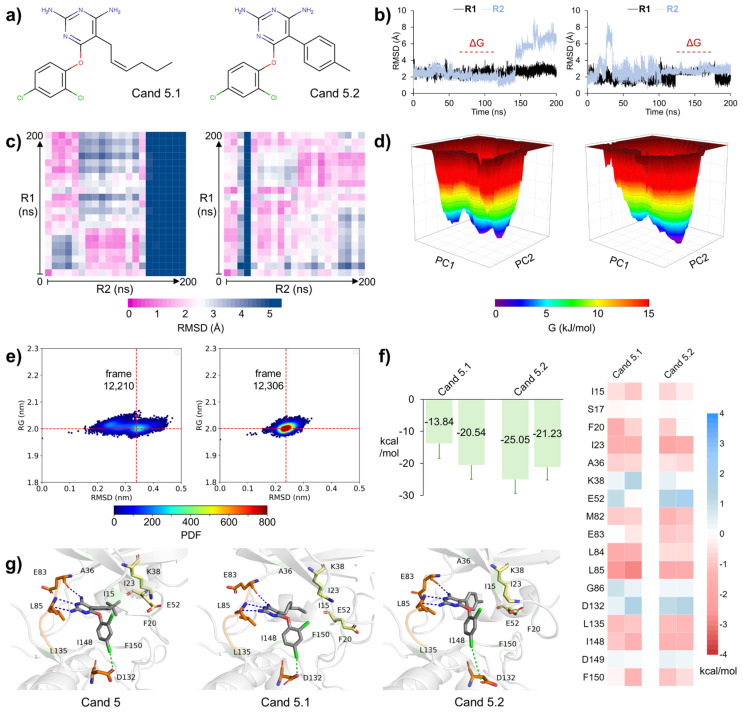
MD assessment of additional members of group 5. (**a**) Two-dimensional structures of Cand 5.1 and 5.2. (**b**) RMSD of ligands across two independent replicates (R1 and R2). Dashed lines indicate the subinterval used for ΔG calculations. (**c**) RMSD matrices comparing ligand conformations between the two independent replicates. (**d**) Principal-component-based free energy landscapes for CK1ε bound to Cand 5.1 and 5.2. (**e**) Probability density function plots for CK1ε complexes with Cand 5.1 and 5.2. (**f**) ΔG for CK1ε/Cand 5.1 and 5.2 complexes, along with per-residue decomposition of the enthalpic contribution. (**g**) Representative ligand conformations from the lowest-energy replicate. CK1ε is shown as a grey cartoon, with the hinge region colored orange and hydrophobic-interacting residues green. Ligands are depicted in grey. E83, L85, and D132 are shown as orange sticks, while K38 and E52 are yellow. Blue and green dashed lines indicate hydrogen and halogen bonds, respectively.

Given the promising binding affinity of Cand 5, we evaluated the binding properties of the two related compounds from group 5, naming them Cand 5.1 and Cand 5.2 (Figure 9a). Cand 5.1 showed an RMSD of 2.55 ± 0.36 Å in R1, indicating a stable binding mode. However, its RMSD increased to 3.34 ± 1.89 Å in R2, mainly due to instability at the end of the simulation. In contrast, Cand 5.2 exhibited RMSD values of 2.08 ± 0.55 Å in R1 and 2.83 ± 0.99 Å in R2, suggesting consistent stability (Table S4, Figure 9b).

The ligand conformations explored by Cand 5.1 showed a stable binding mode in R1. In contrast, R2 exhibited a progressive RMSD increase during the final quarter of the simulation, indicating a conformational rearrangement. This outcome suggests the existence of alternative binding configurations for Cand 5.1 within the CK1ε binding pocket, which may require longer MD simulations to be fully characterized. On the other hand, Cand 5.2 adopted a unique set of conformations across both MD replicates (Figure 9c).

The FEL of the CK1ε/Cand 5.1 complex revealed that the protein explored multiple energy minima, likely reflecting the ligand’s weaker binding. Conversely, the binding of Cand 5.2 stabilized the protein within a more well-defined energy minimum (Figure 9d). Moreover, the tendency of Cand 5.1 to form a more flexible interaction with CK1ε was further supported by its PDF plot, which lacked a clearly predominant conformation. In contrast, Cand 5.2 exhibited a well-defined high-density region, resembling those observed for the reference compounds and Cand 5 (Figure 9e).

The average ΔGs for the CK1ε/Cand 5.1 complex were −13.84 kcal/mol in R1 and −20.54 kcal/mol in R2. In contrast, ΔGs for CK1ε/Cand 5.2 were −25.05 kcal/mol and −21.23 kcal/mol in R1 and R2, respectively (Table S5). The per-residue decomposition trend observed for Cand 5 was maintained in Cand 5.1 and Cand 5.2, with two exceptions. E83 contributed favorably to Cand 5 and Cand 5.2 but had an almost null contribution to Cand 5.1 binding. Meanwhile, D149 was unfavorable in Cand 5 but had a negligible impact on Cand 5.1 and Cand 5.2. (Figure 9f). Interestingly, compounds of group 5 formed halogen bonds with D132 in the C-lobe (Figure 9g), which was reflected as a notable energy contribution in the per-residue decomposition maps. Given their favorable ΔGs and residue-interaction profile, we propose Cand 5 and Cand 5.2 as enhanced-affinity CK1ε inhibitors.

## 3. Discussion

CK1ε has emerged as a promising target for the development of antineoplastic drugs [6,22,23,43,44]. The pharmacological targeting of CK1ε suppresses tumor growth [22,26,45,46] and modulates Wnt signaling pathways [7,47,48]. These effects have been linked to the modulation of oncogenic signaling pathways, apoptosis induction [45,49], reduced cancer cell proliferation [23,50], and increased sensitivity to targeted therapies [22,51,52].

Multiple CK1ε inhibitors have been identified by using pharmacophore models [53,54,55]. However, those models mainly target the buried region of the catalytic pocket, often neglecting the solvent-exposed region. We addressed this limitation by modifying a previously reported pharmacophore to enable the identification of heterocyclic compounds, expanding the structural diversity of the potential inhibitors. In addition, we sought to enhance the ligand interaction with the hinge region of CK1ε by searching for compounds that could form three hydrogen bonds with such a region, in contrast to other reported inhibitors, which form two hydrogen bonds. We expected that this improved engagement would contribute to greater binding stability, as reported [56]. Finally, by prioritizing compounds with a high oral bioavailability potential and pH-dependent pharmacophore compliance, we concentrated on compounds with translational potential.

As expected, we found structural diversity in the compounds of interest. We performed MD analysis only on one of the members of each structural group, considering that the results obtained for the evaluated candidates likely reflect the behavior of the entire group from which they were selected. This extrapolation is supported by the fundamental premise of structure–activity relationships [57,58,59]. To manage computational costs, we selected one representative compound per structural group for molecular dynamic simulations. This strategy assumes that structurally similar compounds share comparable binding modes, in line with structure–activity relationship principles. However, this simplification is one of the limitations of this study, as structurally similar compounds can also exhibit markedly different activities, a phenomenon widely discussed under the concept of activity cliffs [60,61]. For example, Daoud and Taha systematically identified activity cliffs among kinase inhibitors using matched molecular pair analysis, revealing that even small chemical changes can result in substantial potency variations [62]. Therefore, while this approach is efficient, we acknowledge its limitations in fully capturing all activity variations within each group. Thus, further analyses of the non-sampled compounds could still identify potential CK1ε inhibitors.

In MD simulations, we confirmed that the analyzed compounds form the targeted trident-like interaction with the hinge region of CK1ε, involving one hydrogen bond with E83 and two with L85. Similarly, inhibitors displaying this interaction with E83 and L85 of CK1δ, the closest homolog of CK1ε, have been previously reported [63]. Furthermore, these inhibitors have been co-crystallized within CK1δ (PDB: 5IH5, 5IH6), suggesting that this kind of interaction is beneficial for target binding. Notably, three out of eight representative compounds from the identified structural groups displayed ΔGs within the range found for the REF compounds, indicating that the employed strategy is adequate for the intended purpose.

On the other hand, Cand 5, which contains a 2,4-diaminopyrimidine core, showed a more favorable ΔG than the reported inhibitors used as references. Compounds derived from this moiety had been identified as effective kinase inhibitors with in vitro anticancer activities [64,65,66]. Additionally, other 2,4-diaminopyrimidine compounds have been reported to exhibit inhibitory activity against CK1δ (IC_50_ = 0.11–30.00 µM) [67]. Those inhibitors align with six of the seven features in the pharmacophore model, suggesting that this scaffold could be prioritized in the design of new CK1ε inhibitors.

Both K38 and E52, located at the bottom of the pocket, play key roles in the binding of the identified compounds. Previous studies reported an intramolecular interaction between these residues in the apo protein, occurring 46.04% of the time, which decreased to 37.34% upon REF 1 binding [30]. Our findings suggest that K38 can have either a favorable or an unfavorable role in ligand binding, depending on the nature of the substituents buried in the pocket. It contributes favorably to the binding of ligands with polar substituents (Cand 1, Cand 8) but unfavorably when interacting with ligands featuring predominantly hydrophobic substituents (Cand 2–7). Altogether, these results suggest that K38 is a critical determinant of ligand binding, while E52 contributes unfavorably, likely due to its competition with the ligand for interaction with K38.

The dihalogenated rings of group 5 candidates were oriented toward the solvent-exposed region. Their binding to CK1ε was stabilized by halogen bonds with D132 and I23, forming a favorable intermolecular interaction not initially targeted by the employed pharmacophore. These interactions should be considered desirable, especially since few ligands have been reported to interact with the solvent-exposed region of the ATP pocket of CK1ε. Halogens are known to play a crucial role in enhancing binding affinity and specificity due to their ability to engage in halogen bonding, which can further stabilize the ligand–protein complex. This is particularly important for achieving high selectivity within kinase families, as the incorporation of halogens can significantly influence the electrostatic and steric interactions of the ligand. Interestingly, previous studies on polyhalogenated benzimidazole derivatives reported the role of halogens in inhibiting several members of the CK1 and CK2 families, such as CK1ε [68]. The ability of halogens to form stable interactions in the solvent-exposed regions suggests a potential optimization for future ligand design, emphasizing their value in targeting kinases like CK1ε.

## 4. Materials and Methods

### 4.1. Preparation of the CK1ε/REF 1–3 Complexes

The structure of CK1ε in complex with REF 1 was obtained from the RCSB Protein Data Bank (PDB ID: 4HNI). Chain A of CK1ε was isolated from the homodimer, and any unresolved fragments were modeled using the GapRepairer server (http://gaprepairer.cent.uw.edu.pl (accessed on 13 February 2020)) [69]. In contrast, the CK1ε/REF 2–3 complexes were created through molecular docking, following a previously established protocol [38], which was validated by reproducing the crystallographic conformation of the REF 1 co-crystallized with CK1ε. In brief, ligands were docked using a flexible-ligand, rigid-receptor approach within a 10 Å sphere centered on the CK1ε ATP-binding site. A total of 12 combinations of three search algorithms and four scoring functions were used, generating 96 poses. The representative binding mode was selected from the largest cluster.

### 4.2. Virtual Screening

Both the original and modified pharmacophore models were utilized as queries in Pharmit [39] to screen the PubChem database. In brief, the original pharmacophore model was created by analyzing the key intermolecular interactions identified in MD simulations of known CK1ε inhibitors. Eight features were selected based on their frequency, including hydrogen bond donors and acceptors associated with Leu85 and Glu83, aromatic, and hydrophobic interactions involving Phe20, Ala36, Pro66, Met82, Leu135, Ile148, and Lys38. The modified pharmacophore model is described above. Conformers matching the pharmacophore and fitting the CK1ε ATP-binding site were selected. Minimization with Smina [41] allowed for retention of those with a heavy-atom RMSD ≤ 2.0 Å.

The ADME properties and compliance with Lipinski’s Ro5 [42] were assessed using the SwissADME server (http://www.swissadme.ch (accessed on 15 October 2024)) [70]. The compound’s Simplified Molecular Input Line Entry System (SMILES) were generated from the explicit hydrogen-restored structures and used as input. The protonation states were calculated using ChemAxon Marvin Sketch (v23.12.0), while the Bemis–Murcko frameworks [71] were generated in DataWarrior (v06.01.03) [72].

### 4.3. MD Simulations

The CK1ε/Cand complexes were generated by aligning the pharmacophore-matching conformers and subsequently minimizing them as previously described.

MD simulations were conducted for the CK1ε/ligand complexes using GROMACS 2021.6 [73] with the CHARMM36m and CHARMM general force fields [74]. The protein parameters were obtained from chain A of CK1ε utilizing the CHARMM-GUI PDB Reader & Manipulator (https://www.charmm-gui.org/?doc=input/pdbreader (accessed on 20 April 2024)) [75]. Histidine residues were assigned to the neutral tautomer HSE [76]. Ligand parameters were generated using the CHARMM-GUI Ligand Reader & Modeler (https://www.charmm-gui.org/?doc=input/ligandrm (accessed on 6 March 2025)) [77], with protonation states set to reflect the predominant microspecies at pH 7.4.

Simulations were conducted in a periodic cubic box with a dimension of 75 Å to ensure that the complex remained fully solvated and sufficiently distant from the boundaries throughout the simulation. The system contained TIP3P water and neutralizing ions (Na^+^/Cl^−^ at a concentration of 0.15 M). Energy minimization was performed using the steepest-descent algorithm, followed by equilibration in a constant Number of particles, Volume, and Temperature (NVT) ensemble for 125,000 steps at 310.15 K, utilizing a velocity rescaling thermostat [78]. Production simulations were carried out for 200 ns in a constant Number of particles, Pressure, and Temperature (NPT) ensemble, comprising 100 million steps with a 2 fs timestep. A Nosé-Hoover thermostat algorithm [79] and Parrinello-Rahman barostat algorithm [80] were employed to maintain a reference temperature of 310.15 K and pressure of 1 bar.Trajectories were stored every 10 ps.

### 4.4. MD Analysis

The RMSDs were calculated relative to the minimized structures, using backbone atoms for the protein and heavy atoms for the ligands. Additionally, the RMSF was determined for Cα atoms, using the most stable simulation replicate for each system, identified based on the lowest RMSD standard deviation.

DCCMs were generated for the protein Cα atoms using the complete trajectories and the calc_correlation.py script from the MD-TASK suite [81]. On the other hand, the first two principal components used for constructing the free energy landscapes were derived from the eigenvectors and eigenvalues of the protein backbone atoms, obtained by diagonalizing the covariance matrix using gmx covar and gmx anaeig. Finally, FELs were computed with gmx sham and visualized using OriginPro (v2025).

PDF plots were generated based on the complexes Rg and RMSD, calculated using gmx gyrate and gmx rms, respectively. The estimated probability densities were computed via a kernel density estimation using the Geo-Measures plugin [82]. Among the described analyses, DCCM and FEL were performed on the most stable simulation replicate for each system, while PDF plots were generated from the lowest-energy replicates.

The intermolecular interactions for the representative binding modes were predicted using the PLIP server [83] and examined alongside the per-residue decomposition of binding energy.

### 4.5. MM/PBSA Calculation 

The binding free energy (ΔG = ΔH – TΔS) was calculated over a stable 50 ns subinterval of the simulations. The MM/PBSA [84] method was employed to evaluate enthalpic contributions, while Interaction Entropy (IE) [85,86] was employed to estimate entropic contributions. The per-residue decomposition analysis included residues that were within 6 Å of the ligand. All calculations were performed using gmx_MMPBSA [87].

## 5. Conclusions

Casein kinase 1 epsilon (CK1ε) plays a central role in oncogenic signaling pathways and is a promising but understudied target in cancer therapy. However, the lack of selective CK1ε inhibitors has limited therapeutic progress. In this study, we aimed to identify new potential 2,4-diaminopyrimidine-based CK1ε inhibitors through a pharmacophore-based screening approach combined with molecular docking and molecular dynamic simulations. We developed a refined pharmacophore model and used it to search the PubChem database, identifying six structurally diverse compounds predicted to bind the ATP-binding site of CK1ε with favorable stability. Among them, two compounds emerged as the most promising candidates, demonstrating favorable binding stability and free energy values comparable to reference inhibitors. Binding energy calculations supported their potential as effective inhibitors. These findings provide new candidate molecules for further in vitro validation and highlight a viable computational strategy for discovering novel CK1ε inhibitors. Future work will focus on an experimental evaluation and chemical optimization to enhance selectivity and efficacy.

## Data Availability

The original data presented in the study are openly available in Zenodo at DOI 10.5281/zenodo.15066748 and 10.5281/zenodo.15092929.

## Supplementary Materials

The following supporting information can be downloaded at: https://www.mdpi.com/article/10.3390/ph18050741/s1, Table S1: Drug-likeness evaluation of selected compounds; Table S2: Percentage of microspecies with HBD–HBA–HBD configuration at pH 7.4 for selected compounds; Table S3: Scoring comparison of selected compounds in complex with CK1ε and CK1δ; Table S4: Averages and standard deviations of protein and ligand RMSDs for candidates and reference compounds over the 200 ns MD simulations; Table S5: Averages and standard deviations of CK1ε/ligand binding energies; Figure S1: Similarity matrices for the compounds identified with the reported and modified pharmacophore model; Figure S2: Dynamic cross-correlation maps for CK1ε residues across MD simulations; Figure S3: Alternative binding modes of REF 3 and Cand 6; Figure S4: Alpha-carbon RMSF of CK1ε residues.

## Abbreviations

The following abbreviations are used in this manuscript:

CK1	Casein kinase 1
CK1ε	Casein kinase 1 epsilon
CK1δ	Casein kinase 1 delta
IC_50_	Half maximal inhibitory concentration
FDA	Food and Drug Administration
Ro5	Lipinski’s rule of five
ATP	Adenosine triphosphate
PI3Kδ	Phosphoinositide 3-kinase-δ
RMSD	Root-mean-square deviation
HBD	Hydrogen bond donor
HBA	Hydrogen bond acceptor
B&M	Bemis and Murcko
MD	Molecular dynamics
RMSF	Root-mean-square fluctuation
ns	Nanosecond
MM/PBSA	Molecular Mechanics Poisson–Boltzmann Surface Area
IE	Interaction entropy
SMILES	Simplified Molecular Input Line Entry System
ΔG	Change in Gibbs free energy
