# Supplementary material for: In Silico Identification of 2,4-Diaminopyrimidine-Based Compounds as Potential CK1ε Inhibitors
Source: Pharmaceuticals (Basel). 2025 May 17;18(5):741. doi: 10.3390/ph18050741 (PMC12114667; doi:10.3390/ph18050741)
Supplement: Supplementary file 1 [file pharmaceuticals-18-00741-s001.zip › pharmaceuticals-3583187-supplementary.pdf]

Supplementary information

Table S1. Drug-likeness evaluation of selected compounds.

CID	MW	HBA	HBD	MLogP	Ro5 violations
154131101	403.31	3	2	3.54	0
165344163	338.36	5	2	1.55	0
154097017	361.23	3	2	3.68	0
67333797	392.49	4	2	2.81	0
122420865	358.44	3	2	3.31	0
117929859	364.83	3	2	3.35	0
42598499	387.84	4	2	3.03	0
10854465	337.76	3	2	2.68	0
56991138	338.23	4	2	2.42	0
117929842	378.86	3	2	3.58	0
42598501	408.26	4	2	3.30	0
42598502	452.71	4	2	3.42	0
117929872	386.79	5	2	3.89	0
42598029	442.71	4	2	3.80	0
42598503	387.84	4	2	3.03	0
117929827	386.79	5	2	3.89	0
117929317	392.43	5	2	3.15	0
10642998	372.21	3	2	3.18	0
66816113	368.43	2	4	2.30	0
42598034	408.26	4	2	3.30	0
146216232	402.35	7	2	3.58	0
42598730	442.71	4	2	3.80	0
146216222	418.35	8	2	3.31	0
117929635	350.80	3	2	3.12	0
117930011	395.26	3	2	3.24	0
10639478	321.31	4	2	2.57	0
69234051	298.34	4	2	1.15	0
117929866	368.80	4	2	3.51	0
14956530	417.91	3	2	4.10	0
117929806	359.43	3	2	2.55	0
4643995	409.92	3	2	3.21	0
122420887	368.80	4	2	3.51	0
118905651	322.36	4	2	1.69	0
117929569	408.88	4	2	3.26	0
42598500	403.84	5	2	2.51	0
146216217	377.42	4	2	2.93	0
70671725	374.36	6	3	2.44	0
117929964	368.80	4	2	3.51	0
146216224	368.80	4	2	2.57	0
117800553	394.43	4	3	2.20	0
146216218	396.39	6	2	2.51	0
117929902	386.79	5	2	3.89	0
117929857	382.82	4	2	3.73	0
122420872	391.44	4	2	3.15	0
67330664	353.36	6	3	0.75	0
67333567	353.36	6	3	0.75	0
163281030	486.45	8	4	1.52	0

163281029	487.46	9	5	1.52	0
158737408	363.30	2	2	3.42	0
163280449	484.43	8	3	2.57	0
154093371	353.25	3	2	3.60	0
42598033	403.84	5	2	2.51	0
117929588	430.38	8	2	2.37	0
117929483	396.39	6	2	2.78	0
67332209	353.36	6	3	0.75	0
147239201	477.50	7	2	1.18	0
67330728	370.36	6	3	1.75	0
67330674	370.36	6	3	1.75	0
117929930	400.81	5	2	4.11	0
69434957	485.64	6	2	3.52	0
163280975	456.47	6	5	1.85	0
91398045	423.39	7	2	3.57	0
309162	320.39	3	2	2.47	0
146216233	400.36	7	2	2.53	0
91426550	445.39	10	3	2.00	0
71535004	455.47	6	3	1.73	0
163727936	409.35	3	2	4.58	1
76286976	507.97	4	3	3.94	1
90232774	507.97	4	3	3.94	1
90232873	562.93	8	3	3.79	1
131876711	591.44	7	4	3.01	1
144656236	576.96	8	3	3.98	1
420186	612.48	9	3	2.33	1
90232687	526.95	6	3	3.33	1
90232689	526.95	6	3	3.33	1
163281162	508.50	7	4	3.00	1
420023	576.43	7	3	2.96	1
131876683	591.44	7	4	3.01	1
420196	605.47	7	4	3.20	1
57651500	534.87	10	3	4.35	2
139976433	503.55	7	3	4.52	2
123687424	653.76	9	7	−0.61	2
59020580	649.73	9	6	−0.23	2
59020527	649.73	9	6	0.17	2
123552721	653.76	9	7	−0.20	2
136775251	546.50	11	4	4.16	3

CID: PubChem compound identifier, MW: Molecular Weight, HBA: Hydrogen bond acceptor, HBD: Hydrogen bond donor, MLogP: Moriguchi LogP, Ro5: Rule of 5.

Table S2. Percentage of microspecies with HBD-HBA-HBD configuration at pH 7.4 for selected compounds.

CID	HBD-HBA-HBD microspecies [%]	CID	HBD-HBA-HBD microspecies [%]
42598499	100.00	117929588	66.76
56991138	100.00	146216222	65.60
42598501	100.00	146216232	62.46
42598502	100.00	117929866	62.35
42598029	100.00	117929859	62.23
42598503	100.00	117929872	62.20
42598034	100.00	117929483	61.88

42598730	100.00	117929930	61.87
42598500	100.00	117930011	61.75
42598033	100.00	117929635	61.70
10854465	99.96	117929964	61.56
10642998	99.96	117929827	61.33
10639478	99.96	117929857	61.22
67333797	99.50	122420887	60.68
67330728	99.50	117929806	60.62
67330674	99.50	146216233	60.61
67330664	99.49	117929902	60.18
67333567	99.49	122420872	60.14
67332209	99.49	117929317	59.76
118905651	99.06	146216217	59.60
66816113	98.96	117929842	59.52
158737408	98.95	117929569	59.42
309162	98.93	122420865	59.24
70671725	98.86	91426550	5.20
146216218	98.67	117800553	0.00
146216224	98.59	163281030	0.00
154131101	98.22	163281029	0.00
154097017	98.21	163280449	0.00
154093371	97.93	147239201	0.00
69234051	77.68	69434957	0.00
4643995	74.17	163280975	0.00
14956530	74.14	91398045	0.00
165344163	69.85	71535004	0.00

CID: PubChem compound identifier.

Table S3. Scoring comparison of selected compounds in complex with CK1ε and CK1δ.

CID	Docking score on CK1ε [kcal/mol]	Docking score on CK1δ [kcal/mol]
10639478	−8.14	−9.04
10642998	−4.97	−5.45
10854465	−7.37	−9.22
118905651	−8.35	−8.04
146216218	−2.39	−4.84
146216224	−6.92	−6.21
154093371	−7.10	−7.76
154097017	−9.76	−8.75
154131101	−7.91	−9.11
158737408	−7.68	−7.15
309162	−8.33	−8.62
42598029	−7.19	−9.56
42598033	−7.03	−9.07
42598034	−7.76	−9.33
42598499	−6.29	−9.18
42598500	−5.04	−8.90
42598501	−7.24	−9.08
42598502	−6.82	−9.14
42598503	−7.04	−9.48
42598730	−7.82	−9.69
56991138	−6.45	−7.17
66816113	−9.09	−9.95
67330664	−5.01	−4.62
67330674	−9.19	−4.49
67330728	−9.19	−4.49
67332209	−5.08	−4.07
67333567	−5.01	−4.62
67333797	−1.94	−4.18
70671725	−8.72	−8.69

CID: PubChem compound identifier.

Table S4. Averages and standard deviations of protein and ligand RMSD for candidates and reference compounds over the 200 ns MD simulations.

Label	CID	R1 protein RMSD [Å]	R1 ligand RMSD [Å]	R2 protein RMSD [Å]	R2 ligand RMSD [Å]
REF 1	53472153	2.12 ± 0.24	1.68 ± 0.33	2.30 ± 0.34	1.91 ± 0.45
REF 2	78333500	2.25 ± 0.26	4.16 ± 0.46	2.04 ± 0.26	4.52 ± 0.60
REF 3	72950888	2.51 ± 0.32	4.74 ± 0.27	2.47 ± 0.42	2.68 ± 0.47
Cand 1	10854465	2.38 ± 0.35	2.22 ± 0.53	2.00 ± 0.28	1.75 ± 0.28
Cand 2	67333567	2.60 ± 0.39	2.80 ± 0.39	2.40 ± 0.39	2.44 ± 0.40
Cand 3	158737408	2.00 ± 0.27	1.87 ± 0.35	2.15 ± 0.29	1.83 ± 0.41
Cand 4	146216218	2.56 ± 0.31	2.40 ± 0.39	2.53 ± 0.34	2.47 ± 0.58
Cand 5	154131101	2.50 ± 0.36	2.83 ± 0.36	2.27 ± 0.28	2.38 ± 0.40
Cand 5.1	154093371	2.05 ± 0.29	2.55 ± 0.36	2.59 ± 0.47	3.34 ± 1.89
Cand 5.2	154097017	1.83 ± 0.19	2.08 ± 0.55	2.11 ± 0.30	2.83 ± 0.99
Cand 6	66816113	2.36 ± 0.36	3.89 ± 0.80	2.40 ± 0.40	2.67 ± 0.76
Cand 7	67333797	2.22 ± 0.29	22.25 ± 6.95	2.26 ± 0.39	6.56 ± 1.79
Cand 8	309162	1.95 ± 0.28	2.29 ± 1.08	2.38 ± 0.36	2.31 ± 0.49

CID: PubChem compound identifier.

Table S5. Averages and standard deviations of CK1ε/ligand binding energies.

Ligand	REP	ΔH ± SD [kcal/mol]		−TΔS ± SD [kcal/mol]		ΔG ± SD [kcal/mol]	
REF 1	R1	−25.69	3.62	6.09	2.37	−19.60	4.33
	R2	−25.73	3.07	3.70	1.34	−22.03	3.35
REF 2	R1	−30.10	3.88	13.38	4.05	−16.72	5.61
	R2	−28.03	3.23	9.74	7.04	−18.29	7.75
REF 3	R1	−30.79	4.44	6.50	3.61	−24.29	5.72
	R2	−30.22	3.36	7.68	3.43	−22.55	4.81
Cand 1	R1	−19.90	3.52	5.96	2.48	−13.94	4.31
	R2	−20.52	3.26	3.50	1.80	−17.01	3.72
Cand 2	R1	−18.13	3.67	12.25	7.68	−5.88	8.52
	R2	−16.33	4.14	8.72	4.21	−7.61	5.90
Cand 3	R1	−22.23	3.38	2.64	1.41	−19.59	3.66
	R2	−25.03	4.01	3.89	3.00	−21.14	5.01
Cand 4	R1	−23.30	3.68	5.38	2.08	−17.92	4.23
	R2	−26.45	2.96	8.94	4.35	−17.50	5.26
Cand 5	R1	−31.74	4.37	6.19	2.03	−25.54	4.82
	R2	−29.54	2.87	7.23	5.95	−22.31	6.61
Cand 5.1	R1	−21.31	4.25	7.47	1.85	−13.84	4.63
	R2	−24.24	3.50	3.70	2.70	−20.54	4.42
Cand 5.2	R1	−29.30	3.20	4.25	2.84	−25.05	4.28
	R2	−26.34	3.49	5.12	1.85	−21.23	3.95
Cand 6	R1	−26.22	3.88	6.63	3.61	−19.59	5.30
	R2	−24.91	3.70	5.89	2.02	−19.02	4.21
Cand 8	R1	−26.03	3.11	5.94	2.97	−20.09	4.30
	R2	−24.75	3.07	3.88	1.68	−20.87	3.50

REP: Independent MD simulation.

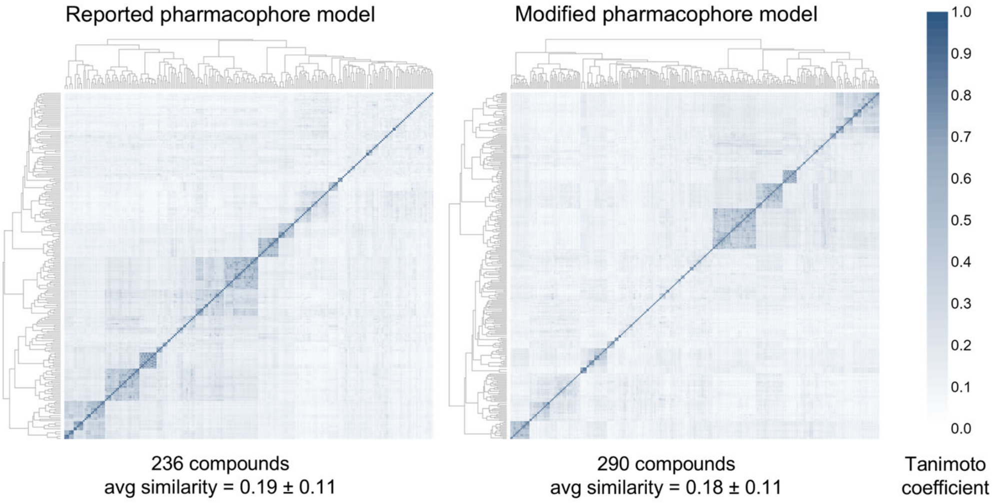

Figure S1. Similarity matrices for the compounds identified with the reported and modified pharmacophore model. Compounds are organized based on a dendrogram model.

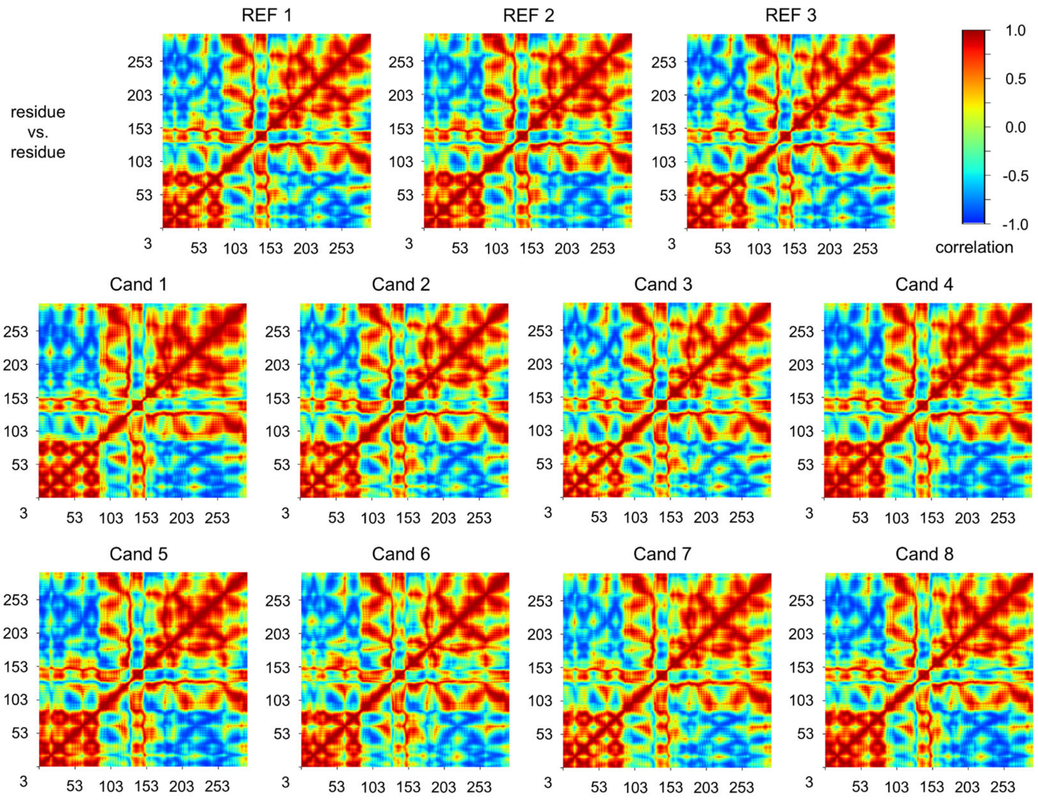

Figure S2. Dynamic cross-correlation maps for CK1ε residues across MD simulations.

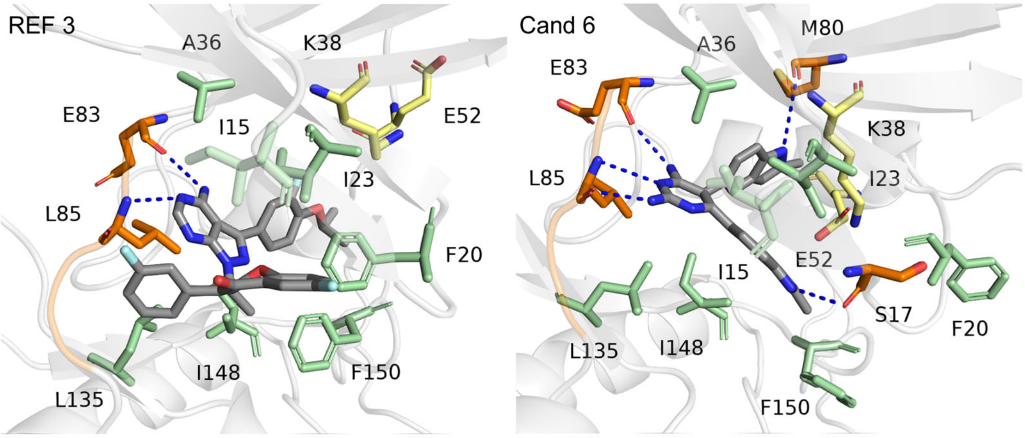

Figure S3. Alternative binding modes of REF 3 and Cand 6. CK1ε is shown as a grey cartoon, with the hinge region colored orange. Ligands are depicted in grey. Interacting residues are shown as sticks; orange for residues involved in hydrogen bonds, green for those participating in hydrophobic interactions, and yellow for K38 and E52. Blue dashed lines indicate hydrogen bonds.

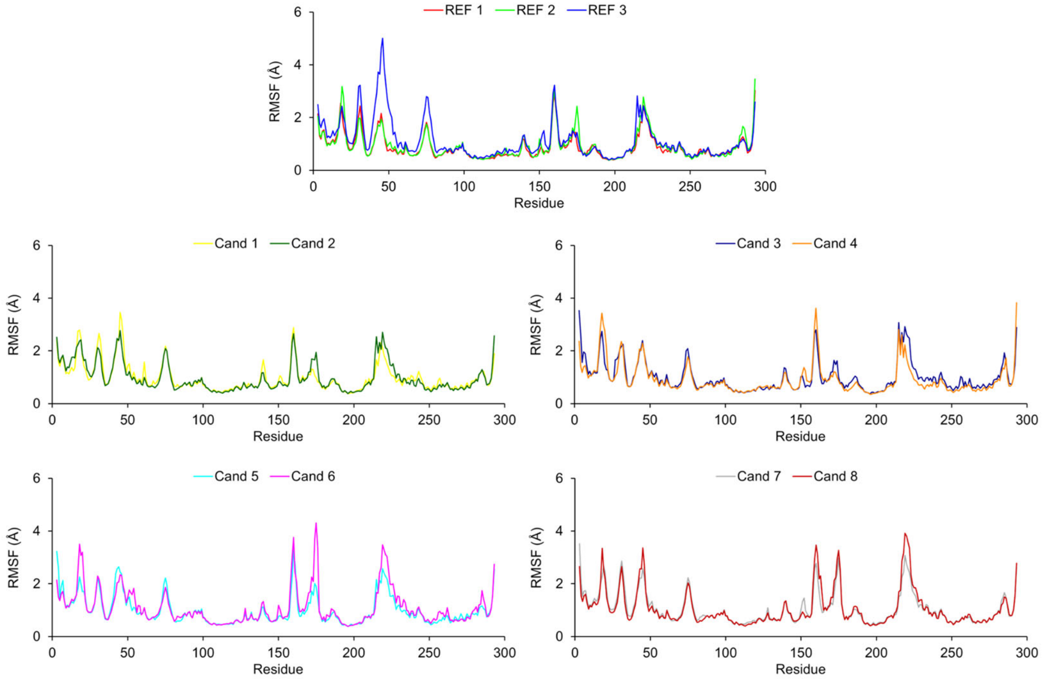

Figure S4. Alpha-carbon RMSF of CK1ε residues. Candidates are displayed in pairs for clarity.
